# Urinary Estrogen Metabolites and Long-Term Mortality Following Breast Cancer

**DOI:** 10.1093/jncics/pkaa014

**Published:** 2020-03-02

**Authors:** Tengteng Wang, Hazel B Nichols, Sarah J Nyante, Patrick T Bradshaw, Patricia G Moorman, Geoffrey C Kabat, Humberto Parada, Nikhil K Khankari, Susan L Teitelbaum, Mary Beth Terry, Regina M Santella, Alfred I Neugut, Marilie D Gammon

**Affiliations:** p1 Department of Epidemiology, University of North Carolina, Chapel Hill, NC, USA; p2 Channing Division of Network Medicine, Department of Medicine, Brigham and Women's Hospital and Harvard Medical School, Boston, MA, USA; p3 Department of Epidemiology, Harvard T.H. Chan School of Public Health, Boston, MA, USA; p4 Department of Radiology, University of North Carolina, Chapel Hill, NC, USA; p5 Division of Epidemiology, University of California, Berkeley, CA, USA; p6 Department of Community and Family Medicine, Duke University, Durham, NC, USA; p7 16 Bon Air Ave, New Rochelle, NY 10804, USA; p8 Division of Epidemiology and Biostatistics, San Diego State University, San Diego, CA, USA; p9 Division of Epidemiology, Vanderbilt University Medical Center, Nashville, TN, USA; p10 Department of Environmental Medicine and Public Health, Icahn School of Medicine at Mount Sinai, New York, NY, USA; p11 Department of Epidemiology, Columbia University, New York, NY, USA; p12 Department of Environmental Health Sciences, Columbia University, New York, NY, USA; p13 Department of Medicine, Columbia University, New York, NY, USA

## Abstract

**Background:**

Estrogen metabolite concentrations of 2-hydroxyestrone (2-OHE_1_) and 16-hydroxyestrone (16-OHE_1_) may be associated with breast carcinogenesis. However, no study has investigated their possible impact on mortality after breast cancer.

**Methods:**

This population-based study was initiated in 1996–1997 with spot urine samples obtained shortly after diagnosis (mean = 96 days) from 683 women newly diagnosed with first primary breast cancer and 434 age-matched women without breast cancer. We measured urinary concentrations of 2-OHE_1_ and 16-OHE_1_ using an enzyme-linked immunoassay. Vital status was determined via the National Death Index (n = 244 deaths after a median of 17.7 years of follow-up). We used multivariable-adjusted Cox proportional hazards to estimate hazard ratios (HRs) and 95% confidence intervals (CIs) for the estrogen metabolites-mortality association. We evaluated effect modification using likelihood ratio tests. All statistical tests were two-sided.

**Results:**

Urinary concentrations of the 2-OHE_1_ to 16-OHE_1_ ratio (>median of 1.8 vs ≤median) were inversely associated with all-cause mortality (HR = 0.74, 95% CI = 0.56 to 0.98) among women with breast cancer. Reduced hazard was also observed for breast cancer mortality (HR = 0.73, 95% CI = 0.45 to 1.17) and cardiovascular diseases mortality (HR = 0.76, 95% CI = 0.47 to 1.23), although the 95% confidence intervals included the null. Similar findings were also observed for women without breast cancer. The association with all-cause mortality was more pronounced among breast cancer participants who began chemotherapy before urine collection (n = 118, HR = 0.42, 95% CI = 0.22 to 0.81) than among those who had not (n = 559, HR = 0.98, 95% CI = 0.72 to 1.34; *P*_interaction_ = .008).

**Conclusions:**

The urinary 2-OHE_1_ to 16-OHE_1_ ratio may be inversely associated with long-term all-cause mortality, which may depend on cancer treatment status at the time of urine collection.

Breast cancer remains the most common cause of cancer death for women worldwide (1). Accumulating evidence indicates that estrogens may have wide-ranging effects on tumor progression (2–5) because of their hormonal stimulation of cell proliferation (6). However, evidence linking endogenous estrogen to survival after breast cancer is limited and inconsistent (5,7).

Examining urinary estrogen metabolites may shed light on the unclear association between both exogenous and endogenous estrogen and mortality after breast cancer. The parent endogenous estrogens, estradiol and estrone, are metabolized along three major irreversible pathways including 2-hydroxyestrone (2-OHE_1_), 4-hydroxyestrone (4-OHE_1_), and 16-hydroxyestrone (16-OHE_1_) (8). Most studies investigating estrogen metabolites and breast cancer incidence have focused on 2-OHE_1_ and 16-OHE_1_, because these two pathways are competitive and mutually exclusive (9). These two metabolites vary in their estrogenic and genotoxic potential: 16-OHE_1_ has strong effects related to cell proliferation and oxidative stress and is considered carcinogenic (9,10); conversely, 2-OHE_1_ has limited estrogenic effects and is possibly antiestrogenic (11,12). Given this, Bradlow et al. (13) proposed that the ratio of 2-OHE_1_ to 16-OHE_1_, which measures the balance between the two competing pathways, may be a biomarker of breast cancer risk. Seven of 15 previous epidemiologic studies have reported that the risk of developing breast cancer was statistically significantly associated with lower levels of the 2-OHE_1_ to 16-OHE_1_ ratio and 2-OHE_1_ and/or higher urinary levels of 16-OHE_1_ (12,14–19).

The urinary estrogen metabolites collected close to the time of breast cancer diagnosis may be associated with prognosis after breast cancer through the progression mechanisms of cell proliferation and oxidative stress (20). However, no study has investigated the role of estrogen metabolites on either overall or cause-specific mortality. Given that previous studies have suggested that cardiovascular disease (CVD) is the most common cause of noncancer death for breast cancer survivors (especially after 5–8 years of diagnosis) (21–23), we specifically considered CVD mortality as one of our study outcomes. Furthermore, investigators have raised concerns about the potential modifying role of menopausal hormone therapy (MHT), obesity, menopausal status, tumor treatment, and breast cancer status on metabolite concentrations levels (12,14–19). A better understanding of the role of estrogen metabolites on mortality outcomes, and the potential modifying effect of these lifestyle and clinical factors on urinary estrogen metabolite levels, may provide clues to health-care providers on refining clinical interventions and follow-up care while taking into account markers of estrogen metabolism.

In our study reported here, first we examined the associations of 2-OHE_1_, 16-OHE_1_, and their ratio, with all-cause, breast cancer, and CVD-specific mortality among women with breast cancer. Second, we explored whether these associations differed by the lifestyle and clinical factors. We also incorporated a comparison group of women without breast cancer to explore whether breast cancer status may modify metabolites-mortality associations.

## Methods

### Study Design

We used the resources from the Long Island Breast Cancer Study Project (LIBCSP), which is a population-based study that included 1508 women with breast cancer and an age-matched cohort of 1556 women without breast cancer who were residents of Nassau and Suffolk counties, Long Island, New York (24). The details of the original case-control (24) and follow-up designs (23) have been previously described. Written, signed informed consent was obtained from participants, and the study procedures were approved by the institutional review boards from all participating institutions.

### Study Population

The analytic cohort for the study reported here consisted of LIBCSP participants with available data on urinary estrogen metabolites. Women with and without breast cancer (n = 1403, 93.0% and n = 1296, 83.3%, respectively) provided a spot urine sample (12). Of those with spot urine samples, a subset (33%) was randomly selected to be assayed for the estrogen metabolites. Additionally, the estrogen metabolite assay was performed on all urine samples from women with in situ tumors (n = 218) and from all African Americans (n = 28) who were not selected as part of the random sample. In sum, the estrogen metabolism assay was performed on a total of 1121 samples (687 women with breast cancer and 434 women without breast cancer). The final analytical sample contained 683 women with breast cancer and 432 women without breast cancer.

### Urinary Estrogen Metabolite Assessment

A 25 ml spot urine sample was obtained by LIBCSP trained field staff at the baseline interview (mean = 96 days after diagnosis for women with breast cancer) (24). Respondents also were asked to complete a checklist of estrogen-related exposures in the past 48 hours (food, medications, and alcohol) and whether breast cancer treatment was initiated in the 6 months prior to urine collection. Urine samples were stored at -80°C in the laboratory of Dr.Regina Santella at Columbia University [according to a standard protocol (25)] for estrogen metabolite measurement via the advanced enzyme-linked immunoassay (Immunacare, Inc, Bethlehem, PA). The laboratory assay’s coefficient of variabilities was 10%, and the intraclass correlation coefficients ranged from 80% to 95% (26,27). All individual metabolites were further normalized to creatinine [urinary estrogen metabolites (ng)/urinary creatinine (mg)] to control for the difference in urine flow rate. More details about the immunoassay were published previously (12).

### Covariate Assessment

The covariate information was primarily drawn from the LIBCSP baseline interviewer-administered questionnaire. These detailed, structured instruments included assessments of sociodemographic characteristics, reproductive and menstrual history, lifestyle factors, medical and medication history, and other factors at or prior to the date of diagnosis for women with breast cancer and date of identification for women without breast cancer (24,28). Diet was self-reported using a modified semiquantitative Block Food Frequency Questionnaire. At the follow-up interview, which occurred approximately 5 years postdiagnosis, the first course of treatment for the first primary breast cancer was self-reported among 1098 breast cancer patients, and medical records were reabstracted for 598 patients. Kappa (κ) coefficients comparing self-report and medical records were high (>90%) (23). Thus, the self-reported treatment modalities were used in this analysis.

### Outcome Assessment

Vital status and date and cause of death were determined via the National Death Index (NDI) (29,30). For women identified as deceased, three event indicators were determined (23): 1) breast cancer–related death, 2) CVD-related death, and 3) death from any cause. For the analyses focused only on women with breast cancer, we used the NDI follow-up through December 31, 2014 (median follow-up = 17.7 years, range = 0.41–18.4 years) and identified 244 all-cause deaths, of which 84 were related to breast cancer and 83 were related to CVD. For our analyses that included both women with and without breast cancer, we used the NDI follow-up through December 31, 2011 (median follow-up = 14.7 years (range = 0.41–15.4 years) and 14.9 years (range = 1.1–15.5 years) for women with and without breast cancer, respectively). The mean time between urine collection and death events was 14.3 years for women with breast cancer and 13.7 years for women without breast cancer.

### Statistical Analysis

Concentrations of the two individual urinary estrogen metabolites (2-OHE_1_ and 16-OHE_1_) and their ratio were dichotomized at the median. Women with metabolite levels no more than median value were chosen as the referent group. We used multivariable Cox proportional hazards regression to estimate hazard ratios (HRs) and 95% confidence intervals (CIs) (31). Person-time of follow-up was calculated from the reference date to the date of death or end date of NDI follow-up (see Outcome Assessment). We assessed the proportional hazards assumption by visual inspections of log(-log(survival))plots. We also tested interactions with follow-up time and each exposure (the two metabolites and their ratio) and also all covariates (in categories; see paragraph below) (31,32). Violations of the proportional hazards assumption were found for age, dietary fat intake, and cholesterol-lowering medications in relation to all-cause mortality among women with breast cancer; thus, corresponding final models included interaction terms between these covariates and follow-up time.

Findings are presented for the three outcomes from four distinct models, with varying adjustment sets. Model 1 includes adjustment for age. Model 2 includes the minimal sufficient adjustment set, which was selected a priori by use of a directed acyclic graph (DAG) (33). This set included age (continuous), education (<high school/high school graduate, college/college graduate, postcollege), total household income (<$15 000–24 999, $25 000–49 999, $50 000–90 000+), oral contraceptive use (ever, never), menopausal hormone use (never, ever), physical activity (never, <0.69, 0.70–2.69, >2.70 hours/week), body mass index (BMI; continuous), cigarette smoking (never, current, past/former), alcohol intake (never, <15, ≥15 g/day), total daily dietary fat intake (continuous), and cholesterol-lowering medications use (never, ever).

Models 3 and 4 were constructed to address the possibility of confounding by cancer treatment. Model 3 included the minimal sufficient adjustment set (from model 2), with additional adjustments for initiation of chemotherapy and/or endocrine therapy prior to urine collection. Model 4 also included the minimal sufficient adjustment set, with further adjustment for the first course of chemotherapy and endocrine therapy for the first primary breast cancer (but with no adjustment for treatment initiated before urine collection). Information on the first course of chemotherapy and endocrine therapy was missing for 28% of women with breast cancer. Thus, we performed multiple imputations by using PROC MI in SAS (34), which is a methodologically sound approach that implements Markov Chain Monte Carlo procedure (35) (25 imputations with 100 iterations) to address bias and loss of precision (36,37).

We evaluated potential effect modification of the urinary metabolite-mortality association among women with breast cancer by history of MHT use (nonuser vs ever-user), BMI (<25 vs ≥25 kg/m^2^), menopausal status (premenopausal vs postmenopausal), smoking history (never vs ever), and chemotherapy (yes vs no) or endocrine therapy initiated before the urine collection (yes vs no). We also considered a model that included both women with and without breast cancer to evaluate modification by breast cancer status (yes vs no). We assessed effect modification on the multiplicative scale (at the α = 0.05 statistical significance level) by comparing nested models with and without the cross-product terms from the likelihood ratio test (38). All statistical tests were two-sided. All analyses were performed using SAS version 9.4 (SAS Institute, Inc, Cary, NC).

## Results

### Study Participant Characteristics

As shown in Table 1, the LIBCSP women with breast cancer included in this study were on average 57.8 years old at diagnosis. Compared with women with breast cancer who had a 2-OHE_1_ to 16-OHE_1_ ratio no more than median value (1.8), women with levels above the median were less likely to be overweight and obese (48.5% vs 60.8%; *P* = .007) but more likely to have smoked cigarettes (57.8% vs 49.3%; *P* = .08) and consumed alcohol (68.6% vs 56.6%; *P* = .008). Other characteristics were similar across levels of the 2-OHE_1_ to 16-OHE_1_ ratio.

**Table 1.** Distribution of selected baseline sociodemographic and disease characteristics among women (with urine sample) diagnosed in 1996–1997 with first primary breast cancer (n = 683), overall and stratified by the ratio of 2-OHE_1_ to 16-OHE_1_ (assessed using urine samples collected an average of 96 days after diagnosis), LIBCSP

Characteristics	All, No. (%) (N = 683)	Ratio ≤ median (1.8), No. (%)(n = 339)	Ratio > median (1.8), No. (%)(n = 344)	*P* ‡
Age at diagnosis, y				
Mean (SD)	57.8 (12.2)	58.1 (12.3)	57.6 (12.2)	.64
Race				
White	635 (93.1)	309 (91.2)	326 (95.0)	.12
Black and other	47 (6.9)	30 (8.8)	17 (5.0)	
Education				
< HS/HS graduate	298 (43.8)	160 (47.5)	138 (40.2)	.16
Some college/college graduate	266 (39.1)	124 (36.8)	142 (41.4)	
Postcollege	116 (17.1)	53 (15.7)	63 (18.4)	
Income				
<$15 000–24 999	129 (18.9)	71 (21.0)	58 (16.9)	.35
$25 000–49 999	201 (29.5)	100 (30.0)	101 (29.5)	
$50 000–>90 000	351 (51.5)	167 (49.4)	184 (53.6)	
Menopausal status				
Premenopausal	236 (35.2)	110 (33.1)	126 (37.2)	.27
Postmenopausal	435 (64.8)	222 (66.9)	213 (62.8)	
Missing	12	7	5	
Oral contraceptive use				
Never	322 (47.3)	183 (54.1)	176 (51.3)	.46
Ever	359 (52.7)	155 (45.9)	167 (48.7)	
Menopausal hormone therapy				
Never	476 (69.7)	245 (72.3)	231 (67.2)	.19
Ever	206 (30.2)	93 (27.4)	113 (32.9)	
Body mass index, kg/m^2^				
<25	306 (45.3)	131 (39.2)	175 (51.5)	.007
25–30	223 (33.1)	117 (35.0)	106 (31.2)	
>30	145 (21.5)	86 (25.8)	59 (17.4)	
Mean (SD)	26.6 (5.6)	27.3 (5.8)	25.8 (5.3)	
Missing	9	5	<5	
Physical activity, h/wk				
None	191 (29.9)	97 (29.9)	94 (29.8)	.91
<0.69	150 (23.5)	77 (23.8)	73 (23.2)	
0.70–2.69	147 (23.0)	71 (21.9)	76 (24.1)	
>2.70	151 (23.6)	79 (24.4)	72 (22.9)	
Missing	44	15	29	
History of active smoking				
None	317 (46.4)	172 (50.7)	145 (42.2)	.08
Current	120 (17.6)	54 (15.9)	66 (19.2)	
Past/former	246 (36.0)	113 (33.3)	133 (38.7)	
Alcohol intake				
None	255 (37.3)	147 (43.4)	108 (31.4)	.008
<15 g/day	327 (47.9)	150 (44.2)	177 (51.5)	
≥15 g/day	101 (14.8)	42 (12.4)	59 (17.1)	
Total daily dietary fat intake, g				
Mean (SD)	52.3 (28.2)	52.2 (28.7)	52.4 (27.2)	.92
Missing	14	<5	10	
Cholesterol-lowering medication				
Yes	71 (10.4)	34 (10.0)	37 (10.8)	.76
No	612 (89.6)	305 (90.0)	307 (89.2)	
Initiated chemotherapy before urine collection*				
Yes	118 (17.4)	62 (18.5)	56 (16.4)	0.50
No	559 (82.6)	274 (81.6)	285 (83.6)	
Missing	6	<5	<5	
Initiated endocrine therapy before urine collection*				
Yes	146 (21.9)	77 (23.3)	69 (20.5)	.45
No	522 (78.1)	254 (76.7)	268 (79.5)	
Missing	15	8	7	
Received first course of chemotherapy for the first primary breast cancer				
Yes	169 (34.6)	86 (36.1)	83 (33.2)	.49
No	319 (65.4)	152 (63.9)	167 (66.8)	
Missing	195	101	94	
Received first course of endocrine therapy for the first primary breast cancer				
Yes	260 (53.5)	99 (42.0)	123 (49.2)	.38
No	226 (46.5)	137 (58.0)	127 (50.8)	
Missing	197	103	94	
Tumor size, cm				.29
≤2	276 (78.4)	156 (78.0)	120 (78.9)	
>2–≤5	65 (18.5)	36 (18.0)	29 (19.1)	
>5	11 (3.1)	8 (4.0)	3 (2.0)	
Missing	331	202	129	
Nodal involvement				.37
No	135 (35.0)	73 (32.3)	62 (38.7)	
Yes	251 (65.0)	153 (67.7)	98 (61.3)	
Missing	297	176	121	
Hormone receptor status†				.40
Negative	82 (21.8)	58 (24.7)	24 (17.0)	
Positive	294 (78.2)	177 (75.3)	117 (83.0)	
Missing	307	167	140	
Invasiveness				.15
In situ	217 (31.8)	99 (29.2)	118 (34.3)	
Invasive	466 (68.2)	240 (70.8)	226 (65.7)	

* Within 6 months before urine sample collection. 2-OHE_1 =_ 2-hydroxyestrone; 16-OHE_1_ = 16-hydroxyestrone; HS = high school; LIBCSP = Long Island Breast Cancer Study Project.

† Hormone receptor positive status defined as either or both estrogen and progesterone receptors is positive; hormone receptor negative status defined as neither the estrogen nor progesterone receptor is positive.

‡ *P* values were calculated from *t* test and χ^2^ test for continuous and categorical variables, respectively. All tests were two-sided.

### Estrogen Metabolite Concentrations

Among women with breast cancer, the 2-OHE_1_ to 16-OHE_1_ ratio did not vary substantially by menopausal status, although the two individual metabolites were lower in postmenopausal women than premenopausal women [as previously reported (12)]. The distribution of the 2-OHE_1_ to 16-OHE_1_ ratio did not differ substantially by history of MHT use (never/ever: 1.9 vs 2.1; *P* = .08), chemotherapy (yes/no: 2.0 vs 2.1; *P* = .62), or endocrine therapy (yes/no: 2.0 vs 1.9; *P* = .39) initiation status at the time of urine collection (Supplementary Figures 1–3, available online).

### All-Cause Mortality

Among women with breast cancer, as shown in Table 2, a 2-OHE_1_ to 16-OHE_1_ ratio above (vs below) the median was associated with reduced risk of all-cause mortality following breast cancer, regardless of the adjustment set included in our models. For example, in model 4, where we adjusted for socioeconomic, lifestyle factors, and first course of chemotherapy and hormone therapy (after performing multiple imputation), hazards were reduced by 26% (HR = 0.74, 95% CI = 0.56 to 0.98). For individual metabolites, in model 4, we observed an inverse association for 2-OHE_1_ (HR = 0.85, 95% CI = 0.63 to 1.14) but a positive association for 16-OHE_1_ (HR = 1.08, 95% CI = 0.79 to 1.44), although all 95% confidence intervals included the null.

**Table 2.** Cox regression hazard ratios and 95% confidence intervals for the association between urinary estrogen metabolites (assessed using urine sample collected approximately 3 months after diagnosis) and all-cause mortality among women diagnosed with first primary breast cancer in 1996–1997 (n = 683) and followed for median of 17.7 years (until December 31, 2014), LIBCSP

Estrogen metabolites	All deaths	PY	All-cause mortality			
			Model 1	Model 2	Model 3	Model 4
			Age-adjusted	Adjusted*	Adjusted†	Adjusted‡
			HR (95% CI)	HR (95% CI)	HR (95% CI)	HR (95% CI)
2-OHE_1_/16-OHE_1_						
≤Median (1.8)	132	4946	1.00 (Referent)	1.00 (Referent)	1.00 (Referent)	1.00 (Referent)
>Median (1.8)	112	5243	0.82 (0.64 to 1.06)	0.83 (0.63 to 1.09)	0.80 (0.61 to 1.06)	0.74 (0.56 to 0.98)
2-OHE_1_ /creatinine, ng/mg						
≤Median (9.9)	133	4812	1.00 (Referent)	1.00 (Referent)	1.00 (Referent)	1.00 (Referent)
>Median (9.9)	101	5008	0.94 (0.72 to 1.22)	0.92 (0.69 to 1.22)	0.88 (0.66 to 1.18)	0.85 (0.63 to 1.14)
16-OHE_1_/creatinine, ng/mg						
≤Median (5.6)	131	4890	1.00 (Referent)	1.00 (Referent)	1.00 (Referent)	1.00 (Referent)
>Median (5.6)	103	4930	1.14 (0.87 to 1.49)	1.07 (0.80 to 1.43)	1.06 (0.79 to 1.42)	1.08 (0.79 to 1.44)

* Model 2 = adjusted for DAG-identified confounders: age, education, total household income, oral contraceptive use, menopausal hormone use, physical activity, BMI, smoking, alcohol intake, total daily dietary fat intake, cholesterol-lowering medications use; model also included interaction between follow-up time with age, total daily dietary fat intake, and cholesterol-lowering medications use. 2-OHE_1 =_ 2-hydroxyestrone; 16-OHE_1_ = 16-hydroxyestrone; BMI = body mass index; CI = confidence interval; DAG = directed acyclic graph; HR = hazard ratio; LIBCSP = Long Island Breast Cancer Study Project; PY = person-years.

† Model 3 = adjusted for DAG-identified confounders + chemotherapy or endocrine therapy before urine collection: age, education, total household income, oral contraceptive use, menopausal hormone use, physical activity, BMI, smoking, alcohol intake, total daily dietary fat intake, cholesterol-lowering medications use, chemotherapy (before urine collection), and endocrine therapy (before urine collection); model also included interaction between follow-up time with age, total daily dietary fat intake, and cholesterol-lowering medications use.

‡ Model 4 = adjusted for DAG-identified confounders + complete course of chemotherapy and endocrine therapy for the first primary breast cancer: age, education, total household income, oral contraceptive use, menopausal hormone use, physical activity, BMI, smoking, alcohol intake, total daily dietary fat intake, cholesterol-lowering medications use, complete course of chemotherapy (after performing multiple imputation), and complete course of endocrine therapy (after performing multiple imputation); model also included interaction between follow-up time with age, total daily dietary fat intake, and cholesterol-lowering medications use.

### Cause-Specific Mortality

As shown in Table 3, among women with breast cancer, breast cancer-specific mortality was consistently reduced for the above the median value of the 2-OHE_1_ to 16-OHE_1_ ratio, regardless of the adjustment set considered. For example, in model 4, where we accounted for both the minimal sufficient adjustment set and first course of treatment (after performing multiple imputation), the hazard was reduced by 27% (HR = 0.73, 95% CI = 0.45 to 1.17). For individual metabolites, all associations were essentially null.

**Table 3.** Cox regression hazard ratios and 95% confidence intervals for the association between urinary estrogen metabolites (assessed using urine samples collected approximately 3 months after diagnosis) and breast cancer-specific and CVD-specific mortality among women diagnosed with first primary breast cancer in 1996–1997 (n = 683) and followed for median of 17.7 years (until December 31, 2014), LIBCSP

Estrogen metabolites	Cause-specific deaths	PY	Cause-specific mortality			
			Model 1	Model 2	Model 3	Model 4
			Age-adjusted	Adjusted*	Adjusted†	Adjusted‡
			HR (95% CI)	HR (95% CI)	HR (95% CI)	HR (95% CI)
Breast cancer–specific mortality						
2-OHE_1_/16-OHE_1_						
≤Median (1.8)	48	4946	1.00 (Referent)	1.00 (Referent)	1.00 (Referent)	1.00 (Referent)
>Median (1.8)	36	5243	0.71 (0.46 to 1.10)	0.77 (0.49 to 1.22)	0.74 (0.46 to 1.19)	0.73 (0.45 to 1.17)
2-OHE_1_ /creatinine, ng/mg						
≤Median (9.9)	41	4812	1.00 (Referent)	1.00 (Referent)	1.00 (Referent)	1.00 (Referent)
>Median (9.9)	43	5008	1.00 (0.64 to 1.56)	1.01 (0.63 to 1.62)	0.95 (0.59 to 1.53)	0.99 (0.61 to 1.62)
16-OHE_1_/creatinine, ng/mg						
≤Median (5.6)	41	4890	1.00 (Referent)	1.00 (Referent)	1.00 (Referent)	1.00 (Referent)
>Median (5.6)	43	4930	1.03 (0.65 to 1.61)	0.99 (0.61 to 1.59)	0.98 (0.59 to 1.60)	1.01 (0.61 to 1.66)
CVD-specific mortality						
2-OHE_1_/16-OHE_1_						
≤Median (1.8)	46	4946	1.00 (Referent)	1.00 (Referent)	1.00 (Referent)	1.00 (Referent)
>Median (1.8)	37	5243	0.78 (0.51 to 1.21)	0.85 (0.53 to 1.35)	0.84 (0.53 to 1.34)	0.76 (0.47 to 1.23)
2-OHE_1_ /creatinine, ng/mg						
≤Median (9.9)	51	4812	1.00 (Referent)	1.00 (Referent)	1.00 (Referent)	1.00 (Referent)
>Median (9.9)	29	5008	0.75 (0.48 to 1.20)	0.70 (0.23 to 1.15)	0.69 (0.42 to 1.14)	0.62 (0.37 to 1.03)
16-OHE_1_/creatinine, ng/mg						
≤Median (5.6)	44	4890	1.00 (Referent)	1.00 (Referent)	1.00 (Referent)	1.00 (Referent)
>Median (5.6)	36	4930	1.32 (0.84 to 2.08)	1.18 (0.73 to 1.92)	1.16 (0.71 to 1.88)	1.19 (0.72 to 1.96)

* Model 2 = adjusted for DAG-identified confounders: age, education, total household income, oral contraceptive use, menopausal hormone use, physical activity, BMI, smoking, alcohol intake, total daily dietary fat intake, and cholesterol-lowering medications use. 2-OHE_1 =_ 2-hydroxyestrone; 16-OHE_1_ = 16-hydroxyestrone; BMI = body mass index; CI = confidence interval; DAG = directed acyclic graph; HR = hazard ratio; LIBCSP = Long Island Breast Cancer Study Project; PY = person-years.

† Model 3 = adjusted for DAG-identified confounders + chemotherapy or endocrine therapy before urine collection: age, education, total household income, oral contraceptive use, menopausal hormone use, physical activity, BMI, smoking, alcohol intake, total daily dietary fat intake, cholesterol-lowering medications use, chemotherapy (before urine collection), and endocrine therapy (before urine collection).

‡ Model 4 = adjusted for DAG-identified confounders + complete course of chemotherapy and endocrine therapy for the first primary breast cancer: age, education, total household income, oral contraceptive use, menopausal hormone use, physical activity, BMI, smoking, alcohol intake, total daily dietary fat intake, cholesterol-lowering medications use, complete course of chemotherapy (after performing multiple imputation), and complete course of endocrine therapy (after performing multiple imputation).

As also shown in Table 3, among women with breast cancer, the 2-OHE_1_ to 16-OHE_1_ ratio was also inversely associated with CVD-specific mortality across different models. For example, the hazard from model 4 was reduced by 24% (HR = 0.76, 95% CI = 0.47 to 1.23). However, for the individual metabolites, the hazards were decreased by 38% for 2-OHE_1_ (model 4: HR = 0.62, 95% CI = 0.37 to 1.03) and increased by 19% for 16-OHE_1_ (model 4: HR = 1.19, 95% CI = 0.72 to 1.96).

### Effect Modification

**Figure 1.**
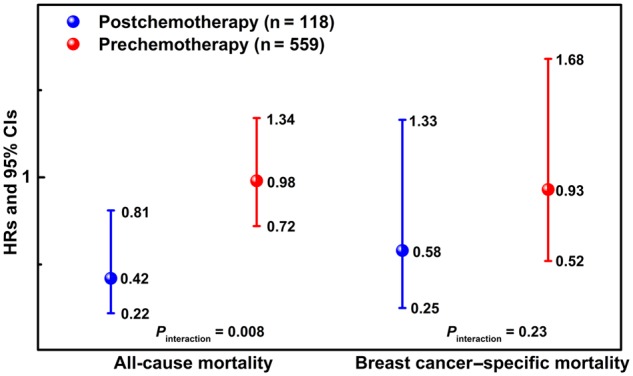
Multivariable-adjusted hazard ratios (HRs) and 95% confidence intervals (CIs) for the association between urinary estrogen metabolites 2-OHE_1_ to 16-OHE_1_ ratio and all-cause and breast cancer–specific mortality among LIBSCP participants diagnosed with breast cancer in 1996–1997 (n = 683), stratified by the timing of urine collection in relation to initiation of chemotherapy. Urine samples were collected approximately 3 months after diagnosis, and breast cancer participants were followed for a median of 17.7 years (until December 31, 2014). Models were adjusted for the DAG-identified adjustment set: age at diagnosis, education, total household income, oral contraceptive use, menopausal hormone use, physical activity, body mass index, smoking, alcohol intake, total daily dietary fat intake, and cholesterol-lowering medications use. 2-OHE_1 =_ 2-hydroxyestrone; 16-OHE_1_ = 16-hydroxyestrone; DAG = directed acyclic graph; LIBCSP = Long Island Breast Cancer Study Project.

As shown in Figure 1, among women with breast cancer, we observed effect modification of the association between the 2-OHE_1_ to 16-OHE_1_ ratio and all-cause mortality by chemotherapy initiated before urine collection. A 2-OHE_1_ to 16-OHE_1_ ratio above (vs below) the median value was strongly associated with a lower all-cause mortality only among women who had received chemotherapy before urine collection (n = 118, HR = 0.42, 95% CI = 0.22 to 0.81) but not among women who had not (n = 559, HR = 0.98, 95% CI = 0.72 to 1.34; *P*_interaction_ = 0.008).

We did not identify any statistically significant effect modification of the associations of the 2-OHE_1_ to 16-OHE_1_ ratio and the two individual metabolites with mortality by history of MHT use (Supplementary Table 1, available online); menopausal status (Supplementary Table 2, available online); BMI (Supplementary Table 3, available online); smoking history (Supplementary Table 4, available online); or endocrine therapy initiated prior to urine collection (Supplementary Table 5, available online).

Where we considered both women with and without breast cancer, the inverse metabolite ratio-mortality associations did not substantially differ by breast cancer status (Supplementary Table 5, available online). However, the magnitude of the hazards among women without breast cancer appeared to be stronger than the corresponding estimate among women with breast cancer, particularly for CVD-specific mortality.

## Discussion

In this population-based study with long-term follow-up, we observed that higher concentration levels of the 2-OHE_1_ to 16-OHE_1_ ratio measured shortly after diagnosis (mean = 96 days) were associated with 18–26% reduced hazards for all-cause mortality among women with breast cancer, regardless of the adjustment set considered. Among women who had initiated chemotherapy before urine collection, the risk reduction was 58%. Higher levels of the 2-OHE_1_ to 16-OHE_1_ ratio were also associated with a 15–29% reduction in breast cancer– and CVD-specific mortality, although the 95% confidence intervals of these estimates included the null value.

A higher 2-OHE_1_ to 16-OHE_1_ ratio has been associated with a lower risk of developing breast cancer in several epidemiologic studies, including our own (12,14–19). However, we are the first to report an inverse association between the 2-OHE_1_ to 16-OHE_1_ ratio and all-cause mortality. Our findings support the hypothesis that the estrogen metabolism pathway favoring 2-hydroxylation over 16-hydroxylation is associated with improved mortality among women with breast cancer. This association is biologically plausible based on the potential mechanism of action proposed for the 2-OHE_1_ to 16-OHE_1_ ratio in relation to breast cancer carcinogenesis and CVD etiology. 16-OHE_1_ is a genotoxic metabolite, which is hypothesized to increase abnormal DNA repair (17), and has direct estrogenic effects on breast cell proliferation, differentiation, and apoptosis because of its high affinity for the estrogen receptor (20,39). In contrast, 2-OHE_1_ may inhibit angiogenesis (40,41) and has low affinity for estrogen receptor (15,20). In addition, previous in vitro studies showed 2-OHE_1_ can inhibit the proliferation of vascular smooth muscle cells, cardiac fibroblasts, and glomerular mesangial cells (42,43). A population-based study has also suggested that the concentration of 2-OHE_1_ and 16-OHE_1_ in urine may be a good predictor of systolic blood pressure (44). Considering that the two pathways are competitive and mutually exclusive, the ratio measure of 2-OHE_1_ to 16-OHE_1_ may be a representative biomarker of an individual’s inherent estrogen metabolism profile (15). Direct epidemiological evidence of circulating estrogen metabolism markers in breast cancer progression is limited, but our findings based on urinary biomarkers are consistent with previous studies using blood-based biomarkers, which reported positive associations of endogenous estrogen and testosterone levels with adverse outcomes among women with breast cancer (5,45,46).

Our finding of a pronounced risk reduction in all-cause mortality in association with a higher 2-OHE_1_ to 16-OHE_1_ ratio among women who had initiated chemotherapy before urine collection was unexpected but biologically plausible. Chemotherapy forms reactive oxygen species and induces oxidative damage for both tumor and normal cells (47). Under such circumstances, a higher 2-OHE_1_ to 16-OHE_1_ ratio may indicate that these individuals may have better antioxidative capacity or they are potentially less susceptible to the toxicity from chemotherapy than those with a lower 2-OHE_1_ to 16-OHE_1_ ratio. Therefore, the biological benefits of a preferred 2-OHE_1 _to 16-OHE_1_ ratio estrogen metabolism profile may become more apparent among women who had already started chemotherapy compared with those who had not at the time of urine collection. However, we cannot rule out that this finding may be due to chance.

Considering previous studies suggested that the 2-OHE_1_ to 16-OHE_1_ ratio level was higher among women without breast cancer than those with breast cancer (12,16,19), we are the first to compare the effects of baseline urinary estrogen metabolites on the risk for cancer and noncancer mortality between a cohort of breast cancer survivors and an age-matched sample of women without breast cancer from the same source population. Although the association between estrogen metabolites and mortality was somewhat stronger among women without breast cancer compared with women with breast cancer, which supported our hypothesis, this difference did not achieve statistical significance. But our results shed light on the potential link of estrogen metabolites and mortality in the general population.

Our study has several limitations. First, we relied on a single measurement of urinary estrogen metabolites from spot urine samples, thus we could not account for any possible longitudinal variability of these biomarkers. However, previous studies have shown relatively good reproducibility and stability of these metabolites (12,48). Second, the lab technique we used is based on conventional enzyme immunoassay, thus we measured only a limited number of metabolites. Recently, a high-performance liquid chromatography-tandem mass spectrometry assay was developed to provide a more accurate, reproducible, and comprehensive assessment of 15 metabolites in multiple pathways (49), including 4-OHE_1_ and others. However, the biological effects of these metabolites are largely unknown. In addition, a previous investigation reported that these metabolites comprised less than 5% of the total estrogen metabolites measured, whereas 2-OHE_1_ and 16-OHE_1_ were the largest at 36% and 38%, respectively (18). Importantly, in the study reported here, we were able to capture two of the three most important estrogen metabolism competing pathways (20). Third, considering that the evidence of factors influencing urinary estrogen metabolites is limited, it is possible that there was residual confounding because of other unrecognized lifestyle or clinical factors (ie, tumor characteristics). Although we have tumor characteristics data (tumor size, nodal involvement, and hormone receptor status) available, we have very limited power to do the sensitivity analysis by adjusting these factors because of the extensive missing data. However, the consistency of our results across models with both parsimonious and comprehensive adjustment sets suggests that our overall finding that urinary estrogen metabolites measured close in time to breast cancer diagnosis may have prognostic significance is likely not due to bias. Finally, one of the issues likely contributing to the imprecision of some of our results is our relatively small sample size, which limited statistical power for stratified analyses. However, to our knowledge, ours is the largest study on this topic to date.

Strengths of our study include evaluation of biologic samples from a well-characterized population-based cohort of women with newly diagnosed first primary breast cancer with long-term follow-up. To our knowledge, ours is the first study to examine urinary estrogen metabolite concentrations in relation to mortality and to explore the modifying role of several key lifestyle and clinical factors. Most previous studies on estrogen metabolites usually excluded a large number of current MHT users (12,14–19). Our results represent the first evidence that history of MHT use, BMI, endocrine therapy, and breast cancer status did not appear to modify the metabolites-mortality association. Future research should weigh the necessity of this exclusion criteria with the benefits of having a larger sample size and the chances for performing more meaningful analyses.

In conclusion, in our population-based study, the estrogen metabolism profile marked by high levels of the ratio of 2-OHE_1_ to16-OHE_1_ is associated with better long-term mortality among women with breast cancer. Our results help elucidate the role of estrogen metabolism on survival after breast cancer and may promote future research in developing routine monitoring programs based on readily accessible noninvasive urinary biomarkers into survivorship research. Future investigations are necessary to confirm our findings and to deepen our understanding of the underlying biological mechanisms for estrogen metabolism–mortality relationships following breast cancer.

## Funding

This work was supported by grants from the National Institutes of Health (U01 CA/ES 66572, R01 CA 66572, P30 CA 013696, P30 ES 009089, and T32 CA 009001) and the Babylon Breast Cancer Coalition.

## Notes

**Role of the funder:** The funders had no role in the design of the study; the collection, analysis, and interpretation of the data; the writing of the manuscript; and the decision to submit the manuscript for publication.

**Disclosures:** AIN has consulted for Otsuka Pharmaceuticals, United Biosource Corporation, Hospira, Teva, and Eisai and is on the Scientific Advisory Board of EHE, Intl. The other authors have no potential conflicts of interest.

Authors' contributions: Study concept and design: all authors; data acquisition: MG, GCK, HP, NKK, SLT, MBT, RMS, and AIN; Statistical analysis: TW in consultation with MG, HBN, SJN, PTB, and PGM; interpretation of data: all authors; drafting of the manuscript: TW and MG; critical revision of the manuscript for important intellectual content: all authors; study supervision: MG; approved the manuscript: all authors.

## Supplementary Material

pkaa014_Supplementary_Data
