# Supplementary material for: Urinary Estrogen Metabolites and Long-Term Mortality Following Breast Cancer
Source: JNCI Cancer Spectr. 2020 Mar 2;4(3):pkaa014. doi: 10.1093/jncics/pkaa014 (PMC7236781; doi:10.1093/jncics/pkaa014)
Supplement: pkaa014_Supplementary_Data [file pkaa014_supplementary_data.pdf]

Supplementary tables

Supplementary Table 1. Multivariable-adjusted hazard ratios and 95% confidence intervals for the association between urinary estrogen metabolites (assessed using urine samples collected ~3 months after diagnosis) and mortality among women diagnosed with first primary breast cancer in 1996-1997 and followed for median of 17.7 years (until December 31, 2014), stratified by menopausal hormone therapy (MHT) use prior to diagnosis, LIBCSP

Characteristics	Menopausal hormone therapy (MHT) status							
	Never users				Ever users			
	Deaths	PY	HR*	95% CI	Deaths	PY	HR*	95% CI
All-Cause Mortality								
$2\text{-}OHE_1/16\text{-}OHE_1 \leq$ Median	105	4,124	1.00	reference	26	1,764	1.00	reference
$2\text{-}OHE_1/16\text{-}OHE_1 >$ Median	72	2,876	0.76	(0.55, 1.04)	40	1,421	1.18	(0.67, 2.07)
p interaction	0.60							
Breast Cancer-Specific Mortality								
$2\text{-}OHE_1/16\text{-}OHE_1 \leq$ Median	38	4,124	1.00	reference	9	1,764	1.00	reference
$2\text{-}OHE_1/16\text{-}OHE_1 >$ Median	27	2,876	0.71	(0.42, 1.20)	9	1,421	0.94	(0.34, 2.59)
p interaction	0.97							
CVD-Specific Mortality								
$2\text{-}OHE_1/16\text{-}OHE_1 \leq$ Median	36	4,124	1.00	reference	10	1,764	1.00	reference
$2\text{-}OHE_1/16\text{-}OHE_1 >$ Median	27	2,876	0.96	(0.56, 1.65)	10	1,421	0.62	(0.22, 2.71)
p interaction	0.94							

*Adjusted for DAG-identified adjustment set: age at diagnosis, education, total household income, oral contraceptive use, menopausal hormone use, physical activity, body mass index, smoking, alcohol intake, total daily dietary fat intake, and cholesterol lowing medications use; model for all-cause mortality also included interaction between follow-up time with age, total daily dietary fat intake, and cholesterol lowing medications use.

Abbreviation: PY-person-year; HR: hazard ratio; CI: confidence interval

Supplementary Table 2. Multivariable-adjusted hazard ratios and 95% confidence intervals for the association between urinary estrogen metabolites (assessed using urine samples collected ~3 months after diagnosis) and mortality among women diagnosed with first primary breast cancer in 1996-1997 and followed for median of 17.7 years (until December 31, 2014), stratified by menopausal status prior to diagnosis, LIBCSP.

Characteristics	Menopausal status							
	Premenopausal				Postmenopausal			
	Deaths	PY	HR*	95% CI	Deaths	PY	HR*	95% CI
All-Cause Mortality								
$2\text{-}OHE_1/16\text{-}OHE_1 \leq$ Median	37	2,175	1.00	reference	119	3,558	1.00	reference
$2\text{-}OHE_1/16\text{-}OHE_1 >$ Median	14	1,590	0.69	(0.38, 1.27)	73	2,669	0.88	(0.65, 1.20)
p interaction	0.62							
Breast Cancer-Specific Mortality								
$2\text{-}OHE_1/16\text{-}OHE_1 \leq$ Median	24	2,175	1.00	reference	33	3,558	1.00	reference
$2\text{-}OHE_1/16\text{-}OHE_1 >$ Median	9	1,590	0.77	(0.36, 1.65)	17	2,669	0.83	(0.46, 1.51)
p interaction	0.76							
CVD-Specific Mortality								
$2\text{-}OHE_1/16\text{-}OHE_1 \leq$ Median	9	2,175	1.00	reference	48	3,558	1.00	reference
$2\text{-}OHE_1/16\text{-}OHE_1 >$ Median	N.A.	1,590	N.A.	N.A.	23	2,669	0.89	(0.54, 1.47)
p interaction	N.A.							

*Adjusted for DAG-identified adjustment set: age at diagnosis, education, total household income, oral contraceptive use, menopausal hormone use, physical activity, body mass index, smoking, alcohol intake, total daily dietary fat intake, and cholesterol lowing medications use; model for all-cause mortality also included interaction between follow-up time with age, total daily dietary fat intake, and cholesterol lowing medications use.
Abbreviation: PY-person-year; HR: hazard ratio; CI: confidence interval; N.A.: not available because of cell size < 5.

Supplementary Table 3. Multivariable-adjusted hazard ratios and 95% confidence intervals for the association between urinary estrogen metabolites (assessed using urine samples collected ~3 months after diagnosis) and mortality among women diagnosed with first primary breast cancer in 1996-1997 and followed for median of 17.7 years (until December 31, 2014), stratified by BMI prior to diagnosis, LIBCSP

Characteristics	BMI							
	BMI < 25 kg/m^2				BMI ≥ 25 kg/m^2			
	Deaths	PY	HR*	95% CI	Deaths	PY	HR*	95% CI
All-Cause Mortality								
2-OHE_1/16-OHE_1 ≤ Median	40	2,003	1.00	reference	90	2,863	1.00	reference
2-OHE_1/16-OHE_1 > Median	48	2,726	0.99	(0.60, 1.63)	64	2,445	0.75	(0.53, 1.07)
p interaction	0.50							
Breast Cancer-Specific Mortality								
2-OHE_1/16-OHE_1 ≤ Median	19	2,003	1.00	reference	29	2,863	1.00	reference
2-OHE_1/16-OHE_1 > Median	15	2,726	0.86	(0.39, 1.92)	21	2,445	0.83	(0.45, 1.52)
p interaction	0.54							
CVD-Specific Mortality								
2-OHE_1/16-OHE_1 ≤ Median	9	2,003	1.00	reference	35	2,863	1.00	reference
2-OHE_1/16-OHE_1 > Median	13	2,726	0.92	(0.35, 2.42)	24	2,445	0.77	(0.44, 1.35)
p interaction	0.41							

*Adjusted for DAG-identified adjustment set: age at diagnosis, education, total household income, oral contraceptive use, menopausal hormone use, physical activity, body mass index, smoking, alcohol intake, total daily dietary fat intake, and cholesterol lowing medications use; model for all-cause mortality also included interaction between follow-up time with age, total daily dietary fat intake, and cholesterol lowing medications use.

Abbreviation: PY-person-year; HR: hazard ratio; CI: confidence interval

Supplementary Table 4. Multivariable-adjusted hazard ratios and 95% confidence intervals for the association between urinary estrogen metabolites (assessed using urine samples collected ~3 months after diagnosis) and mortality among women diagnosed with first primary breast cancer in 1996-1997 and followed for median of 17.7 years (until December 31, 2014), stratified by smoking history, LIBCSP

Characteristics	Smoking history							
	Never				Ever			
	Deaths	PY	HR*	95% CI	Deaths	PY	HR*	95% CI
All-Cause Mortality								
$2\text{-}OHE_1/16\text{-}OHE_1 \leq$ Median	61	2,533	1.00	reference	71	2,413	1.00	reference
$2\text{-}OHE_1/16\text{-}OHE_1 >$ Median	49	2,181	0.84	(0.56, 1.26)	63	3,062	0.75	(0.52, 1.09)
p interaction	0.58							
Breast Cancer-Specific Mortality								
$2\text{-}OHE_1/16\text{-}OHE_1 \leq$ Median	23	2,533	1.00	reference	25	2,413	1.00	reference
$2\text{-}OHE_1/16\text{-}OHE_1 >$ Median	22	2,181	1.20	(0.64, 2.27)	14	3,062	0.46	(0.23, 1.02)
p interaction	0.07							
CVD-Specific Mortality								
$2\text{-}OHE_1/16\text{-}OHE_1 \leq$ Median	28	2,533	1.00	reference	18	2,413	1.00	reference
$2\text{-}OHE_1/16\text{-}OHE_1 >$ Median	17	2,181	0.60	(0.31, 1.16)	20	3,062	1.11	(0.55, 2.23)
p interaction	0.18							

*Adjusted for DAG-identified adjustment set: age at diagnosis, education, total household income, oral contraceptive use, menopausal hormone use, physical activity, body mass index, smoking, alcohol intake, total daily dietary fat intake, and cholesterol lowing medications use; model for all-cause mortality also included interaction between follow-up time with age, total daily dietary fat intake, and cholesterol lowing medications use.

Abbreviation: PY-person-year; HR: hazard ratio; CI: confidence interval

Supplementary Table 5. Multivariable-adjusted hazard ratios and 95% confidence intervals for the association between urinary estrogen metabolites (assessed using urine samples collected ~3 months after diagnosis) and mortality among women diagnosed with first primary breast cancer in 1996-1997 and followed for median of 17.7 years (until December 31, 2014), stratified by endocrine therapy initiated before urine sample collection, LIBCSP

Characteristics	Initiated endocrine therapy before urine sample collection							
	Yes				No			
	Deaths	PY	HR*	95% CI	Deaths	PY	HR*	95% CI
All-Cause Mortality								
$2\text{-}OHE_1/16\text{-}OHE_1 \leq$ Median	32	1,154	1.00	reference	95	3,688	1.00	reference
$2\text{-}OHE_1/16\text{-}OHE_1 >$ Median	27	1,050	0.86	(0.49, 1.49)	83	4,083	0.85	(0.61, 1.17)
p interaction					0.92			
Breast Cancer-Specific Mortality								
$2\text{-}OHE_1/16\text{-}OHE_1 \leq$ Median	30	1,154	1.00	reference	35	3,688	1.00	reference
$2\text{-}OHE_1/16\text{-}OHE_1 >$ Median	22	1,050	0.69	(0.24, 1.98)	26	4,083	0.79	(0.46, 1.36)
p interaction					0.91			
CVD-Specific Mortality								
$2\text{-}OHE_1/16\text{-}OHE_1 \leq$ Median	24	1,154	1.00	reference	34	3,688	1.00	reference
$2\text{-}OHE_1/16\text{-}OHE_1 >$ Median	23	1,050	1.04	(0.44, 2.46)	24	4,083	0.76	(0.43, 1.34)
p interaction					0.52			

*Adjusted for DAG-identified adjustment set: age at diagnosis, education, total household income, oral contraceptive use, menopausal hormone use, physical activity, body mass index, smoking, alcohol intake, total daily dietary fat intake, and cholesterol lowing medications use; model for all-cause mortality also included interaction between follow-up time with age, total daily dietary fat intake, and cholesterol lowing medications use.

Abbreviation: PY-person-year; HR: hazard ratio; CI: confidence interval

Supplementary Table 6. Multivariable-adjusted hazard ratios and 95% confidence intervals for the association between urinary estrogen metabolites (assessed using urine samples collected ~3 months after identification in 1996-1997) and mortality (vital status ascertained through December 31, 2011 for both women with and without breast cancer), stratified by breast cancer status at baseline (N=1,121), LIBCSP

Characteristics		Breast cancer status								
		Women with breast cancer				Women without breast cancer				
		Deaths	PY	HR*	95% CI	Deaths	PY	HR*	95% CI	*p interaction*
All-Cause Mortality										
2-OHE_1/16-OHE_1										
	≤ Median	110	4,289	1.00	reference	44	2,917	1.00	reference	
	> Median	93	4,517	0.84	(0.63, 1.14)	36	3,075	0.82	(0.50, 1.36)	0.85
2-OHE_1/creatinine (ng/mg)										
	≤ Median	114	4,196	1.00	reference	52	2,883	1.00	reference	
	> Median	82	4,290	0.90	(0.66, 1.22)	25	2,966	0.70	(0.40, 1.24)	0.23
16-OHE_1/creatinine (ng/mg)										
	≤ Median	109	4,253	1.00	reference	55	2,860	1.00	reference	
	> Median	87	4,233	1.54	(0.78, 3.03)	22	2,988	1.72	(0.54, 5.53)	0.21
CVD-Specific Mortality										
2-OHE_1/16-OHE_1										
	≤ Median	42	4,289	1.00	reference	27	2,917	1.00	reference	
	> Median	33	4,517	0.82	(0.50, 1.34)	16	3,075	0.62	(0.31, 1.26)	0.91
2-OHE_1/creatinine (ng/mg)										
	≤ Median	47	4,196	1.00	reference	30	2,883	1.00	reference	
	> Median	26	4,290	0.73	(0.41, 1.31)	11	2,966	0.61	(0.27, 1.40)	0.28
16-OHE_1/creatinine (ng/mg)										
	≤ Median	40	4,253	1.00	reference	32	2,860	1.00	reference	
	> Median	33	4,233	1.89	(0.45, 7.76)	9	2,988	3.31	(0.62, 17.87)	0.01

*Adjusted for DAG-identified adjustment set: age at diagnosis, education, total household income, oral contraceptive use, menopausal hormone use, physical activity, body mass index, smoking, alcohol intake, total daily dietary fat intake, cholesterol lowing medications use; model also included interaction between follow-up time with age, total daily dietary fat intake, and cholesterol lowing medications use for all-cause mortality, and interaction of time with age for CVD-specific mortality.

Abbreviation: LIBCSP: Long Island Breast Cancer Study Project; PY-person-year; HR: hazard ratio; CI: confidence interval; CVD: cardiovascular diseases

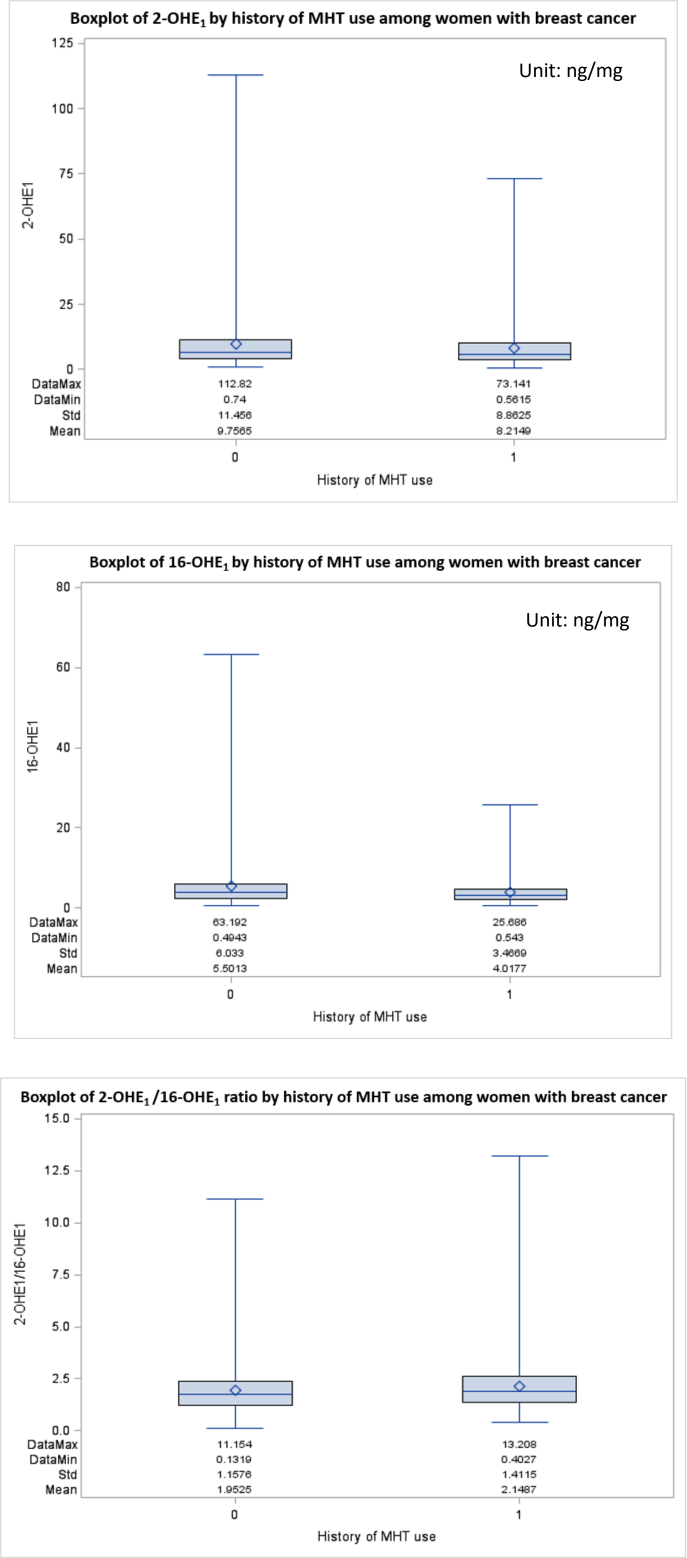

Supplementary Figure 1. Urinary estrogen metabolite concentrations at baseline by MHT use among women with breast cancer (0=never user, 1=ever user)

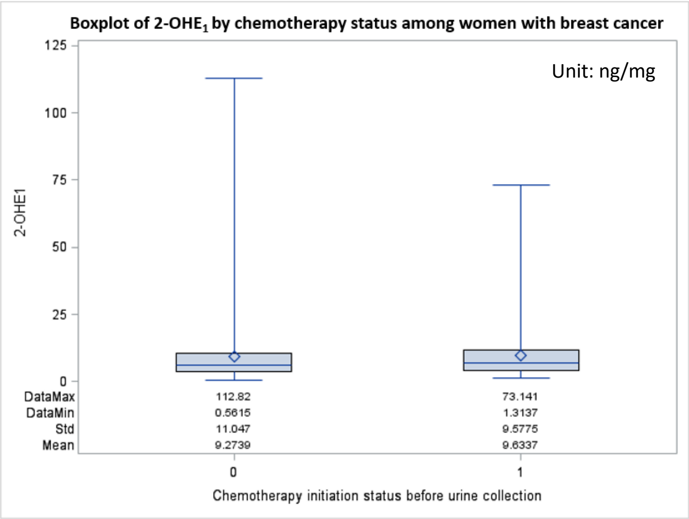

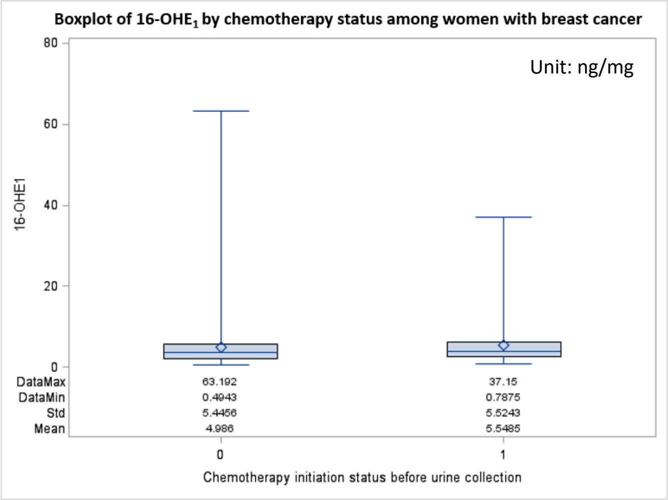

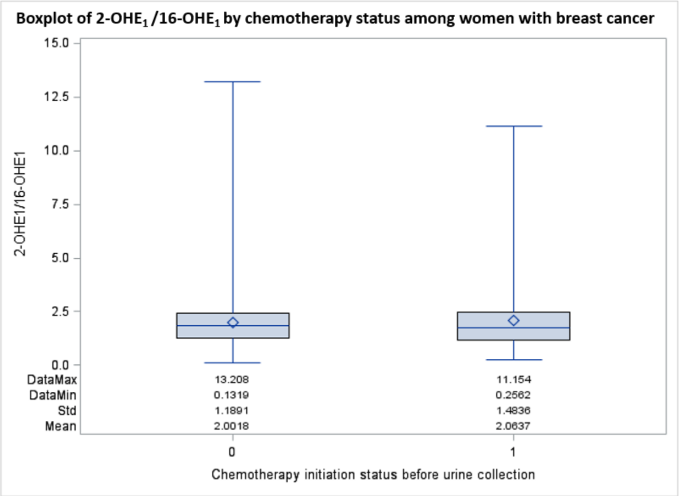

Supplementary Figure 2. Urinary estrogen metabolite concentrations at baseline by chemotherapy initiated before urine collection among women with breast cancer (0=not initiated, 1=initiated)

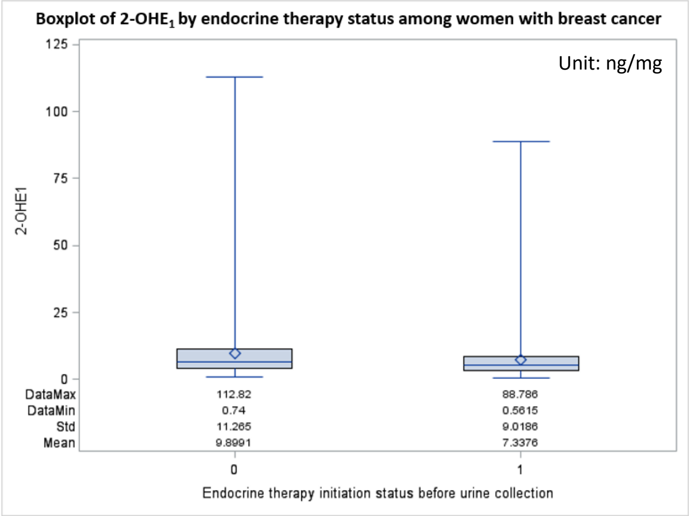

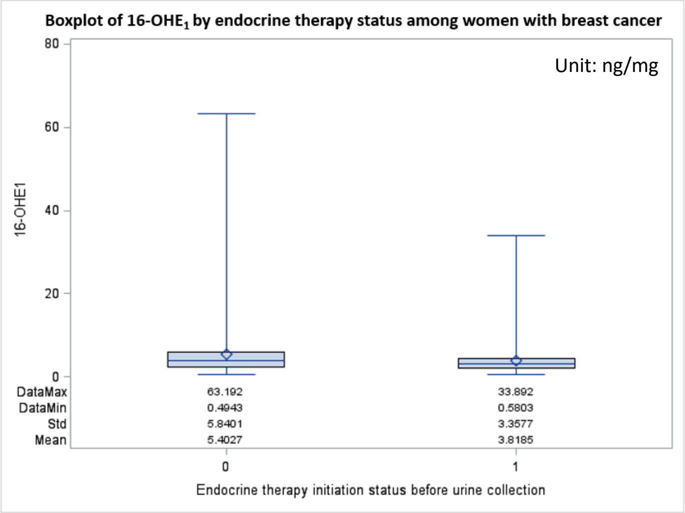

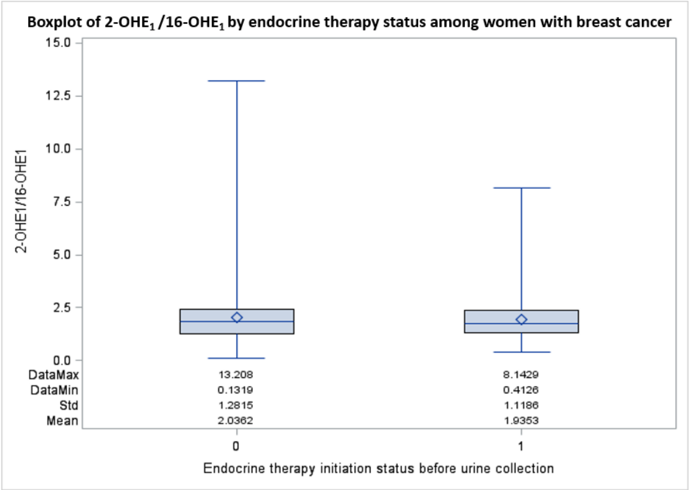

Supplementary Figure 3. Urinary estrogen metabolite concentrations at baseline by endocrine therapy initiated before urine collection among women with breast cancer (0=not initiated, 1=initiated)
